# Sunlight Polymerization of Poly(amidoxime) Hydrogel Membrane for Enhanced Uranium Extraction from Seawater

**DOI:** 10.1002/advs.201900085

**Published:** 2019-04-04

**Authors:** Chunxin Ma, Jinxiang Gao, Dong Wang, Yihui Yuan, Jun Wen, Bingjie Yan, Shilei Zhao, Xuemei Zhao, Ye Sun, Xiaolin Wang, Ning Wang

**Affiliations:** ^1^ State Key Laboratory of Marine Resources Utilization in South China Sea Hainan University Haikou 570228 P. R. China; ^2^ Institute of Nuclear Physics and Chemistry China Academy of Engineering Physics Mianyang 621900 P. R. China

**Keywords:** hydrogel membranes, poly(amidoxime), semi‐interpenetrating networks, sunlight polymerization, uranium extraction from seawater

## Abstract

The uranium level in seawater is ≈1000 times as high as terrestrial ores and can provide potential near‐infinite fuel for the nuclear energy industry. However, it is still a significant challenge to develop high‐efficiency and low‐cost adsorbents for massively extracting uranium from seawater. Herein, a simple and fast method through low‐energy consumption sunlight polymerization to direct fabrication of a poly(amidoxime) (PAO) hydrogel membrane, which exhibits high uranium adsorption capacity, is reported. This PAO hydrogel owns semi‐interpenetrating structure and a hydrophilic poly(acrylamide) 3D network of hydrogel which can disperse and fix PAOs well. As a result, the amidoxime groups of PAOs exhibit an outstanding uranium adsorption efficiency (718 ± 16.6 and 1279 ± 14.5 mg g^−1^ of *m*_uranium_/*m*_PAO_ in 8 and 32 ppm uranium‐spiked seawater, respectively) among reported hydrogel‐based adsorbents. Most importantly, U‐uptake capacity of this hydrogel can achieve 4.87 ± 0.38 mg g^−1^ of *m*_uranium_/*m*_dry gel_ just after four weeks within natural seawater. Furthermore, this hydrogel can be massively produced through low‐energy consumption and environmentally‐friendly sunlight polymerization. This work will provide a high‐efficiency and low‐cost adsorbent for massive uranium extraction from seawater.

Nuclear power is one of the most important alternatives to replace conventional fossil fuels, in response to global increasing energy demands and serious environmental problems. While the proven uranium resources on land are rather limited and can only supply the demand of nuclear energy for about several decades.^1^ However, seawater owns more than 4.5 × 10^9^ ton uranium, which is as ≈1000 times as the amount of terrestrial ores and can provide potential near‐infinite fuel for long‐term sustainable development of nuclear energy industry.^1^ Furthermore, uranium in seawater can be adsorbed through relatively green methods without complicated post‐treatments of uranium‐containing waste.^2^ In the past decades, diverse adsorbing materials for extracting uranium from seawater have been explored including inorganic adsorbents,^3^, ^4^, ^5^, ^6^ porous‐structured adsorbents^7^, ^8^, ^9^, ^10^, ^11^, ^12^ (such as metal−organic frameworks (MOFs),^8^ covalent organic frameworks (COFs),^9^ porous aromatic frameworks (PAFs),^11^ and porous organic polymers (POPs)^12^), organic/polymeric materials,^13^, ^14^, ^15^ protein/biomass‐based materials,^16^, ^17^, ^18^ and so on. But for all this, due to the ultralow concentration (≈3.3 µg L^−1^) of uranium, various competitive ions and complex chemical/biological environment in ocean, it is still a significant challenge to develop high‐efficient adsorbing materials and systems to massively extract uranium from seawater for commercial application.^1^, ^2^, ^19^

Amidoxime‐functionalized polymers (AFPs) are most promising adsorbents for large‐scale extraction of uranium from seawater, because, compared with other adsorbing materials, they can own both high adsorption capacity and good selectivity for uranyl ions (UO_2_^2+^).^19^, ^20^ This kind of AFPs can be designed in various types, such as resins,^21^, ^22^, ^23^ membranes,^24^ fibers,^25^, ^26^, ^27^ and so on, through radiation‐induced graft polymerization (RIGP), atom transfer radical polymerization (ATRP), or chemical/UV‐initiated polymerization. However, because these solid adsorbents of AFPs are naturally dense, so that uranyl ions are commonly difficult to penetrate into the inner part and only the outer amidoxime groups can adsorb uranium effectively.^28^, ^29^ Additionally, the adsorbed uranyl ions can form a cross‐linked polymeric layer, which can also make it much more difficult for other uranyl ions to migrate into the interior part.^28^, ^29^, ^30^ To improve the uranium adsorption capacity and accelerate the rate of AFP adsorbents, there are some strategies to increase the specific surface area of them including fabricating ultrathin, ultrafine, and microporous structure.^7^, ^31^, ^32^ For example, Oyola and Dai^31^ reported a hierarchical microporous‐mesoporous poly(amidoxime) (PAO)‐grafted copolymer from a nanoporous initiator based on ATRP. Compared with common PAO‐grafted copolymers, it can attain high uranium adsorption capacity owing to porous structure and consequently high specific surface area. For another instance, Zhang et al.^32^ developed a nanofiber‐based AFP via a two‐nozzle electrospinning: Both the ultrathin and porous structure of the PAO nanofiber mat can enhance the specific surface area to achieve an effective uranium extraction from seawater. However, the design of high specific surface area often tends to reduce the mechanical strength of AFP adsorbents or make them hard to be massively produced.

Most recently, hydrogel‐based uranium adsorbents have also attracted increasing attention, on account of their good uranium adsorbing performance and relatively simple preparing process.^19^ Hydrogel‐based adsorbents own hydrophilic 3D network containing lots of water, so the uranyl ions can diffuse into each part of the hydrogel and be captured easily.^19^, ^33^, ^34^ For example, hydrogel‐based adsorbents owning amino/carboxyl groups or chitosan structures can achieve relatively high uranium adsorption capacity, while they cannot have good selectivity.^35^, ^36^, ^37^ For another instance, hydrogel‐based adsorbents containing some specific proteins can own ultrahigh selectivity of uranium adsorption, while they are too expensive to be produced massively.^18^, ^38^ Overall, the AFP hydrogels can couple outstanding uranium adsorption capacity and good selectivity.^39^, ^40^ However, as polymers containing nitrile groups commonly cannot dissolve in water, nowadays existing methods need to fabricate organic gels first with grafted/copolymerized nitrile groups, and then transform them into hydrogels.^19^ By these methods, nitrile groups commonly cannot be amidoximated enough and the AFP hydrogels are often inhomogeneous, which seriously limit the uranium adsorption capacity especially from seawater.^19^, ^39^ Additionally, amidoxime‐functionalized monomers or polymers also cannot form hydrogels directly, because they are insoluble in neutral water.

Herein, inspired by embedding fluorescent polymers into semi‐interpenetrating network (semi‐IPN) hydrogel in our previous work,^41^ we developed a convenient method to directly fabricate PAO semi‐IPN hydrogel membrane for high uranium adsorption capacity via eco‐friendly sunlight (UV) polymerization. First of all, because the amidoxime group of the PAO is weakly acidic,^14^ we discovered the PAO can be dissolved in alkaline aqueous solution and monomers can still polymerize under sunlight (UV) to fabricate semi‐IPN hydrogel directly at this condition (**Scheme** 1a). In this as‐prepared hydrogel, the cross‐linked poly(acrylamide) 3D network can disperse and fix a large amount of PAOs at the molecular level and provide good hydrophilic channels for uranyl ions to pass through.^19^, ^20^ Consequently, the amidoxime groups of the PAOs can high‐efficiently chelate uranyl ions (Scheme 1b) and this hydrogel membrane can have excellent capacity of uranium adsorption among reported hydrogel‐based adsorbents. This PAO hydrogel membrane will be a promising high‐efficient adsorbent for massive uranium extraction from seawater.

**Scheme 1:**
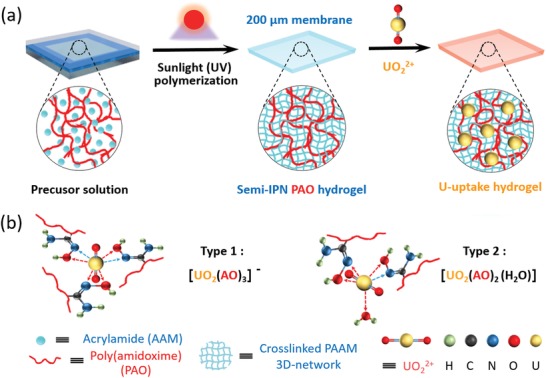
The illustration of producing the PAO semi‐IPN hydrogel and the selective uranyl adsorbing mechanism of them. a) The sunlight (UV) polymerization and uranium uptake of the hydrogel; b) The two main uranyl complexes with amidoxime groups for the uranyl adsorption of the hydrogel from seawater.^20^

The PAO was synthesized through amidoximating the PAN according to the reported literature (Figure S5a, Supporting Information).^19^ As shown in the Fourier transform infrared (FTIR) spectra (**Figure** 1a), the disappearance of the PAN characteristic peak 2244 cm^−1^ (C≡N stretch) and the appearance peaks of C=N (1649 cm^−1^ stretch), N—O (938 cm^−1^ stretch) indicate the nitrile groups of PANs have completely transformed into amidoxime groups of PAOs. X‐ray photoelectron spectroscopy (XPS) spectra of PAO and PAN can also verify the formation of PAO (Figure S6a, Supporting Information): The intense peak at 529.6 eV indicates the formation of N—O—H of PAO. At the same time, the two peaks of PAO at 397.4 and 283.5 eV are almost the same as the two peaks (397.2 and 283.3 eV) of PAN, which indicates the retainment of N1s and C1s, respectively. Additionally, in high‐resolution XPS (Figure S6b,c, Supporting Information), there are obvious change of peak C1s from PAN to PAO. All of these XPS spectra can manifest the C≡N groups have transformed into C=N completely. At the same time, ^13^C‐NMR (Figure S7, Supporting Information) of PAO and the time‐of‐flight mass spectra (TOF‐MS) (Figure S8, Supporting Information) also verify the form of PAO further.

**Figure 1:**
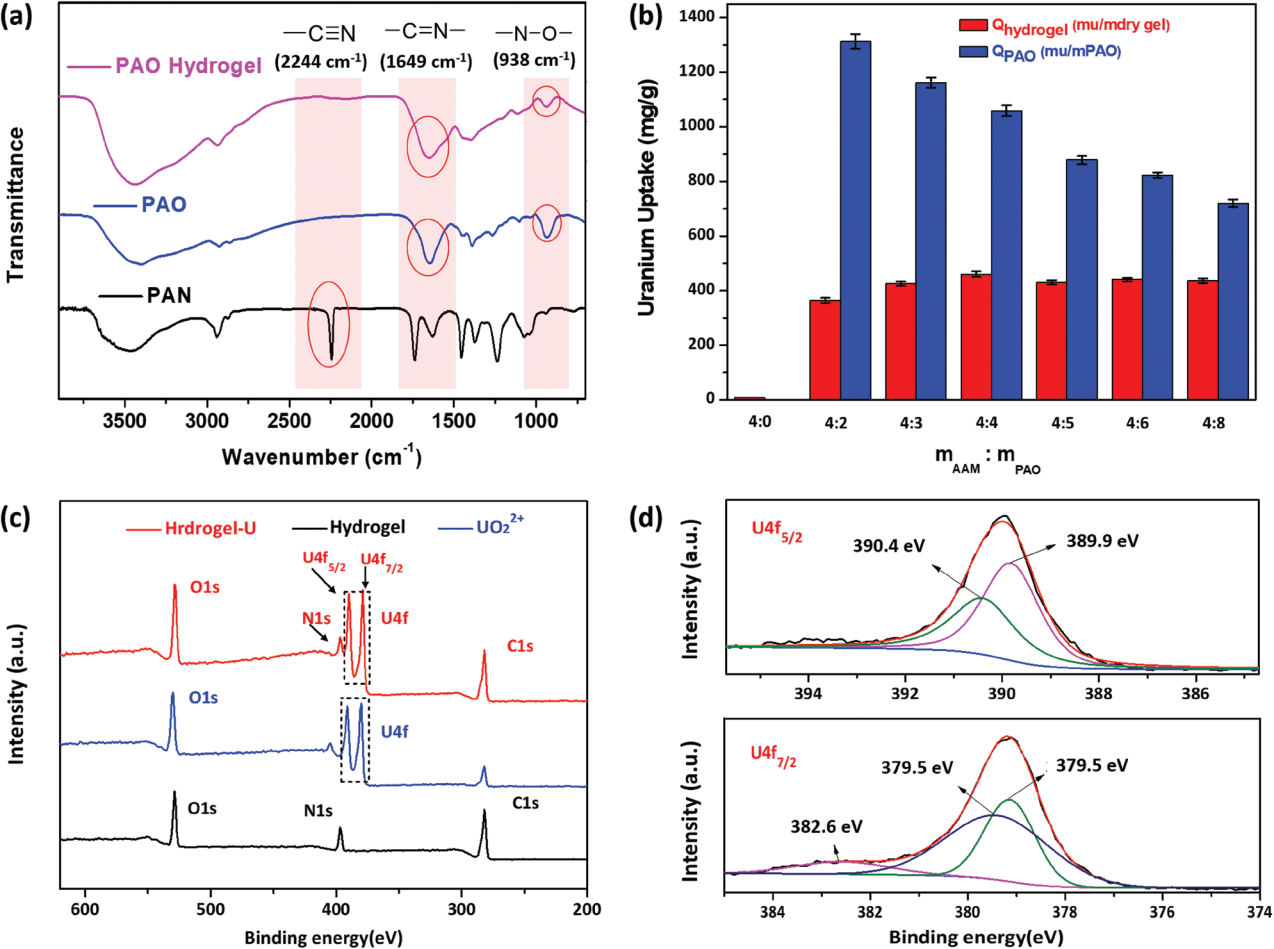
Characterizations of PAN, PAO, PAO hydrogel, and U‐uptake PAO hydrogel. a) The FTIR spectra of PAN, PAO, and PAO hydrogel (*m*_AAM_:*m*_PAO_ = 4:4), respectively. b) The uranium adsorption capacity of different PAO hydrogels in 32 ppm uranium‐spiked seawater for 144 h. c) The survey XPS spectra of U‐uptake PAO hydrogel (*m*_AAM_:*m*_PAO_ = 4:4), PAO hydrogel (*m*_AAM_:*m*_PAO_ = 4:4), and uranyl ion, respectively. d) The high‐resolution XPS spectra of the uranyl ion of the U‐uptake PAO hydrogel (*m*_AAM_:*m*_PAO_ = 4:4).

Different from reported methods which fabricate organic gels first and then transform them into PAO hydrogels, we directly fabricated a PAO hydrogel with semi‐IPN structure through fast sunlight (UV) polymerization (Figure S9, Movie S1, Supporting Information). After added into a 0.3 m NaOH aqueous solution, the amidoxime groups of PAO can change into the hydrophilic negative ions and can be dissolved, which is the key for directly preparing PAO hydrogel. Consequently, the PAOs can be embedded into the cross‐linked poly(acrylamide) 3D network of hydrogel and dispersed at the molecular level through UV‐polymerization (Figure S9a, Movie S1, Supporting Information). Seven PAO hydrogels were prepared with different mass ratios of *m*_AAM_/*m*_PAO_ (4:0, 4:2, 4:3, 4:4, 4:5, 4:6, and 4:8). The precursor solution can form a hydrogel in 15 min via UV‐polymerization under a UV lamp (the UV intensity is 2.2 ± 0.1 mW cm^−2^). Further, under sunlight (the UV intensity is 2.5 ± 0.2 mW cm^−2^) for no more than 10 min, can replace the irradiation of UV lamp to obtain hydrogel well, which can provide a fast, cost‐efficient, and green method for mass production of uranium‐adsorbing materials without expensive equipment (Figure S9b, Movie S1, Supporting Information). As shown in the FTIR spectra (Figure 1a), the 1649 cm^−1^ (C=N stretch) and the 938 cm^−1^ (N—O stretch) specific peaks of the amidoxime groups confirm the PAOs have been fixed in the poly(acrylamide) (PAAM) 3D network. Additionally, this sunlight (UV) polymerization can obtain 200 µm hydrogel membrane with good mechanical properties (Table S3, Supporting Information) and homogeneous structure (Figures S9c and S14, Movie S1, Supporting Information). Compared with common bulk hydrogels, this 200 µm hydrogel membrane can provide high specific surface area and achieve fast uranium adsorption.

As a result, this kind of hydrogel membranes can provide highly enhanced capacity of uranium adsorption. We tested the uranium extraction performance of hydrogels synthesized at different PAO ratios (*m*_AAM_:*m*_PAO_ from 4:0 to 4:8). As shown in Figure 1b and Figure S10 (Supporting Information), compared with the hydrogel (*m*_AAM_:*m*_PAO_ = 4:0) without PAO, which almost cannot adsorb uranium, PAO hydrogels have outstanding uranium adsorption capacity. At the beginning of increasing the proportion of PAO, the uranium adsorption capacity of hydrogel (*Q*_hydrogel_, *m*_U_/*m*_dry gel_) starts to augment; when the ratio of the *m*_AAM_:*m*_PAO_ reaches to 4:4 (the *Q*_hydrogel_ achieves the highest value), continue increasing the proportion of PAO, the *Q*_hydrogel_ will almost have no change. On the other hand, the uranium adsorption capacity of PAO (*Q*_PAO_, *m*_U_/*m*_PAO_) is always decreased when increasing the proportion of PAO. Because the PAO is more expensive than the AAM, the decrease of PAO uranium adsorption capacity will raise the cost of uranium extraction. Overall, considering the *Q*_hydrogel_ and *Q*_PAO_, the hydrogel with the ratio of *m*_AAM_:*m*_PAO_ = 4:4 may be better than others (Figure 1b). As shown in Figure 1c, uranium adsorption of hydrogel can be verified through XPS spectra. Compared with the original PAO hydrogel, the U‐uptake hydrogel and the specific double peaks (390.5 and 379.2 eV) of uranium‐containing hydrogel can indicate the uranyl ions have been adsorbed in the hydrogel. The high‐resolution XPS spectra (U4f: U4f_5/2_, U4f_7/2_) of the U‐uptake PAO hydrogel further verify the existence of uranyl ions (Figure 1d).

Compared with reported uranium adsorbing materials, especially hydrogel‐based adsorbents, this PAO semi‐IPN hydrogel membrane (200 µm thickness) shows outstanding adsorption capacity in uranium‐spiked seawater. In 32, 16, and 8 ppm uranium‐spiked seawater, the PAO hydrogel (*m*_AAM_:*m*_PAO_ = 4:4) showed good uranium extraction performance (**Figure** 2a). After 24 h, the *Q*_hydrogel_ reached to 353 ± 6.6, 238 ± 4.7, and 170 ± 5.7 mg g^−1^, respectively; after 96 h, the *Q*_hydrogel_ reached to 439 ± 6, 337 ± 4.4, and 246 ± 5.2 mg g^−1^, respectively; finally, after 600 h, the hydrogel membrane can achieve saturated capacities of 556 ± 6.3, 434 ± 4.7, and 312 ± 7.2 mg g^−1^, respectively, which owns the outstanding uranium adsorption capacity among reported hydrogel‐based adsorbents. Furthermore, the *Q*_PAO_ in hydrogel can attain as high as 1279 ± 14.5, 998 ± 10.8, and 718 ± 16.6 mg g^−1^ of *m*_uranium_/*m*_PAO_, in 32, 16, and 8 ppm uranium‐spiked seawater, respectively, which have a ultrahigh uranium adsorption capacity compared with existing hydrogel‐based/membrane‐based adsorbents containing amidoxime groups (Figure S11, Supporting Information). This U‐uptake of hydrogel can also be demonstrated via scanning electron microscopy (SEM) images (Figure 2b). The original SEM image of PAO hydrogel shows relatively big porous structure of about 5–10 µm diameter, after uranium adsorption in 8 ppm uranium‐spiked seawater for 600 h, the SEM image of PAO hydrogel shows smaller porous structure of about 50–100 nm diameter. This change of hydrogel may be owing to the adsorption of uranyl ions which can obtain the further cross‐linkage of hydrogel. At the same time, as shown in Figure S12 (Supporting Information), after uranium adsorption, the degree of porous structure changes in SEM images is also distinctly different between the outer and inner parts of the U‐uptake hydrogel: The porous structure near surface is denser and smaller than the inner's, which may indicate the outer part can adsorb more uranium than the inner part. Furthermore, as shown in Figure S13 (Supporting Information), the SEM images before and after uranium adsorption of PAO hydrogel with different ratios of *m*_AAM_:*m*_PAO_ from 4:2 to 4:8 also show similar change, compared with the hydrogel without PAO (*m*_AAM_:*m*_PAO_ = 4:0) whose SEM image has almost no change. In addition, with the increase of PAO proportion in hydrogel, the porous diameter in SEM became smaller and inhomogeneous.

**Figure 2:**
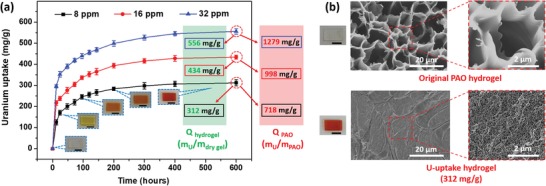
Uranium adsorption capacity of PAO hydrogel membrane (*m*_AAM_:*m*_PAO_ = 4:4) in uranium‐spiked seawater. a) Adsorption kinetics of hydrogel within different uranium concentrations. b) SEM images of the original PAO hydrogel and the U‐uptake hydrogel, respectively. (The photos are hydrogel membrane adsorbing different amounts of uranium. All scale bars in photos are 1.0 cm.)

The impact of pH on the adsorption performance was investigated in uranium‐spiked ultrapure water solution (32 ppm) for 96 h with a pH range of 3.0–8.0. As shown in **Figure** 3a, the uranium adsorption capacity of PAO hydrogel is strongly affected by the pH of solution although each of the PAO hydrogels shows a fast adsorbing process for uranium uptake. The amount of uranium uptake increases significantly from pH 3.0 to 6.0 (from 98 ± 6.7 to 607 ± 9.7 mg g^−1^) and decreases gradually from pH 6.0 to 8.0 (from 607 ± 9.7 to 456 ± 10.5 mg g^−1^). Although the maximum uranium adsorption capacity is achieved at pH ≈ 6.0, at pH ≈ 8.0 (around the pH range of natural seawater), the PAO hydrogel still shows a good adsorption of 456 ± 10.5 mg g^−1^. The fast uranium adsorption process of hydrogel in high uranium concentration (100 ppm) spiked ultrapure water can be observed for no more than 2 h (Movie S2, Supporting Information). In addition, at pH range from 4 to 6, this hydrogel can also be utilized for recovering uranium from acidic wastewater containing uranium.

**Figure 3:**
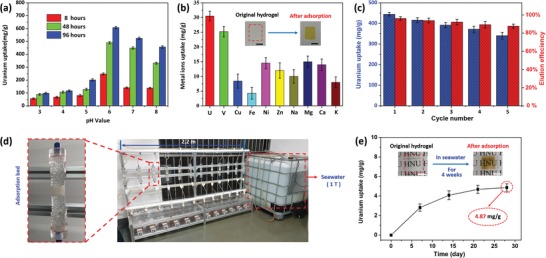
pH dependence, selectivity, and recycle properties of the PAO hydrogel (*m*_AAM_:*m*_PAO_ = 4:4) membrane for uranium extraction. a) The uranium adsorption efficiency of PAO hydrogel in 32 ppm uranium‐spiked ultrapure water solutions with different pH value. b) Adsorption selectivity of PAO hydrogel on the uranyl ion and other metal ions in a simulated seawater (U, V, Cu, Fe, Ni, Zn are as 100 times as the natural seawater; Na, Ca, Mg, K are equal to the natural seawater). c) The uranium adsorption capacities (blue columns) and recovery rates of elution (red columns) in five adsorption–desorption cycles (elution solution of 1.0 m Na_2_CO_3_ and 0.1 m H_2_O_2_). d) The flow‐through uranium extraction system containing 12 parallel adsorption beds with 1.0 T of natural seawater. e) U‐adsorption performance of the PAO hydrogel membrane (*m*_AAM_:*m*_PAO_ = 4:4) from natural seawater during four weeks by the flow‐through uranium extraction system. (All scale bars in photos are 1.0 cm.)

As shown in Figure 3b, the hydrogel adsorption selectivity on different metal ions has been studied. The uranium adsorption capacity is far higher than any other metal ions in the simulated seawater (Table S1, Supporting Information), except that the vanadium uptake is almost as the 80% as the uranium uptake, which indicates a good potential application for massively extracting uranium from seawater based on the outstanding selectivity of uranium adsorption.

As shown in Figure S15 and Movie S3 (Supporting Information), the uranium in the PAO hydrogel membrane (*m*_AAM_:*m*_PAO_ = 4:4) containing 10 mg dry gel can be easily desorbed through 500 mL eluent (mixture solution of 1.0 m Na_2_CO_3_ and 0.1 m H_2_O_2_) for no more than 35 min. As shown in Figure 3c and Figure S5 (Supporting Information), the eluent efficiency of U‐uptake hydrogel can reach to (95.35 ± 4.29)% at the first time; after regeneration, the uranium adsorption capacity of hydrogel at the second time can also reach to (93.51 ± 2.6)% [(415.8 ± 11.6) mg g^−1^] U‐uptake as the first adsorption process [(444.7 ± 9.4) mg g^−1^]. After five adsorption–desorption circles, the hydrogel can still retain the (76.51 ± 3.7)% [(340.2 ± 16.4) mg g^−1^)] uranium adsorption capacity of original PAO hydrogel membrane and the eluent efficiency of uranium can still attain (86.98 ± 5.1)%. At the same time, after five circles, the mechanical properties of PAO hydrogel can also keep well (Table S3, Supporting Information), the strength at break (54.4 ± 3.9 kPa), Young's modulus (137 ± 6.3 kPa), and elongation at break [(38.3 ± 7.7)%] only changed to 57.8 ± 5.6 kPa, 140 ± 5.2 kPa, and [(36.2 ± 8.1)%], respectively. Additionally, there are obvious color changes among the original hydrogel, before and after eluent U‐uptake hydrogel membrane, which can also indicate the fast and high‐efficient uranium adsorption–desorption process (Figure S16 and Movie S3, Supporting Information). Owing to the good property of recyclable uranium adsorption, this PAO hydrogel membrane can be reused for many times in extracting uranium. The uranium extraction from natural seawater has also been tested with a flow‐through system (Figure 3d and Figure S17, Supporting Information). As shown in Figure 3e, the average uranium adsorption capacity can reach to 2.82 ± 0.37, 4.08 ± 0.46, 4.69 ± 0.42, and 4.87 ± 0.38 mg g^−1^ of *m*_uranium_/*m*_dry gel_ after 1, 2, 3, and 4 weeks, respectively, which indicates the PAO hydrogel can provide for a potential high and fast uranium extraction method from seawater.

In conclusion, a simple and fast method for massively fabricating PAO hydrogel membrane‐based adsorbent owing highly enhanced uranium adsorption efficiency has been developed. Through a low‐energy consumption and eco‐friendly sunlight polymerization within alkaline condition, we can fabricate the semi‐IPN PAO hydrogel membrane. The PAO in this hydrogel (*m*_AAM_:*m*_PAO_ = 4:4) can own high efficiency of uranium adsorption (718 ± 16.6 mg g^−1^ in 8 ppm, 1279 ± 14.5 mg g^−1^ in 32 ppm of *m*_uranium_/*m*_PAO_; 312 ± 7.2 mg g^−1^ in 8 ppm, 556 ± 6.3 mg g^−1^ in 32 ppm of *m*_uranium_/*m*_dry gel_) in uranium‐spiked seawater, which has the outstanding U‐uptake capacity among reported hydrogel‐based adsorbents. Additionally, the PAO hydrogel membrane can be reused for at least five circles of uranium adsorption–desorption process and still keep a high uranium adsorption capacity and elution efficiency. Most importantly, in natural seawater, U‐uptake capacity of this PAO hydrogel can also reach to 4.87 ± 0.38 mg g^−1^ of *m*_uranium_/*m*_dry_ just in four weeks. This high‐efficient and low‐cost PAO hydrogel membrane will be a promising adsorbent for large‐scale uranium extraction from seawater. The work will inspire the design and fabrication of other types of high‐efficient uranium adsorbents with semi‐IPN structures, such as fiber‐based, film‐based, and composite materials, as well as hydrogel‐based adsorbents.

## Conflict of Interest

The authors declare no conflict of interest.

## Supporting information

Supplementary

Supplementary

Supplementary

Supplementary
