# Supplementary material for: Sunlight Polymerization of Poly(amidoxime) Hydrogel Membrane for Enhanced Uranium Extraction from Seawater
Source: Adv Sci (Weinh). 2019 Apr 4;6(13):1900085. doi: 10.1002/advs.201900085 (PMC6662065; doi:10.1002/advs.201900085)
Supplement: Supplementary file 1 — Supplementary [file ADVS-6-1900085-s002.pdf]

Copyright WILEY-VCH Verlag GmbH & Co. KGaA, 69469 Weinheim, Germany, 2019.

ADVANCED SCIENCE
Open Access

Supporting Information

for *Adv. Sci.,* DOI: 10.1002/advs.201900085

Sunlight Polymerization of Poly(amidoxime) Hydrogel Membrane for Enhanced Uranium Extraction from Seawater

*Chunxin Ma, Jinxiang Gao, Dong Wang, Yihui Yuan, Jun Wen, Bingjie Yan, Shilei Zhao, Xuemei Zhao, Ye Sun, Xiaolin Wang, and Ning Wang**

Supporting Information *for*

Electronic supplementary information (ESI)

Sunlight-Polymerization of Poly(amidoxime) Hydrogel Membrane for Enhanced Uranium Extraction from Seawater

Chunxin Ma, Jinxiang Gao, Dong Wang, Yihui Yuan, Jun Wen, Bingjie Yan, Shilei Zhao, Xuemei Zhao, Ye Sun, Xiaolin Wang, Ning Wang*

Dr. C Ma, J Gao, Dr. D Wang, Dr. Y Yuan, B Yan, S Zhao, X Zhao, Y Sun, Prof. N Wang State Key Laboratory of Marine Resources Utilization in South China Sea, Hainan University, Haikou 570228, P. R. China
E-mail: wangn02@foxmail.com

Dr. J wen, Prof. X Wang
Institute of Nuclear Physics and Chemistry, China Academy of Engineering Physics,
Mianyang 621900, P. R. China

Chunxin Ma and Jinxiang Gao contributed equally to this work.

Table of Contents

Experimental Section 4

Materials 4

Characterizations 4

Method of detecting uranium element concentration 5

Test of uranium adsorption capacity 5

Test of uranium desorption rate 6

Synthesis of PAO 6

Fabrication of PAO Semi-IPN hydrogel membranes 6

Test of hydrogel adsorption selectivity on different ions 7

Test of hydrogel uranium adsorption from natural seawater 7

Data Analysis 8

Figure S1. The curvilinear regression of uranium concentration-absorbance in the uranium-spiked ultrapure water. 8

Figure S2. The curvilinear regression of uranium concentration-absorbance in uranium-spiked seawater. 8

Figure S3. Comparison of the Arsenazo (III) method and ICP-Mass method for measuring uranium concentration. 9

Figure S4 (a) The curvilinear regression of uranium concentration-absorbance in simulated seawater. (b) Comparison of uranium adsorption capacities between PAO hydrogel in U-uptake seawater and simulated seawater. 10

Figure S5. (a) Synthesis of water-insoluble PAO; (b) Synthesis of water-soluble PAO salt. 11

Figure S6. XPS spectra of PAN and PAO. 11

Figure S7. The ^{13}C-NMR spectra of the (a) PAN and (b) PAO in DMSO-D_6, respectively. 12

Figure S8. Time-of-flight MS of (a) PAN, (b) PAO, (c) alkali-treated PAO), respectively. 13

Figure S9. Process of masiively fabricating of PAO Semi-IPN hydrogel membrane. 14

Figure S10. Uranium adsorption kinetics of PAO hydrogel membranes with different mass ratios of PAO/AAM in 32 ppm uranium-spiked natural seawater. 15

Figure S11. Comparison of adsorption capacities among hydrogel-based and membrane-based adsorbents. 16

Figure S12. SEM images of surface and inner part of U-uptake PAO hydrogel (m_{AAM}/m_{PAO} = 4 : 4, 312 mg/g) 17

Figure S13. SEM images of PAO hydrogels (a) before and (b) after Uranium adsorption with different mass ratios of PAO/AAM. 18

Figure S14. (a) The SEM image of original PAO hydrogel (m_{AAM} : m_{PAO} = 4 : 4). (b) The transparent property of U-uptake PAO hydrogels. 19

Figure S15. Uranium desorption kinetics of PAO hydrogel membranes in the elution solution. 20

Figure S16. The uranium adsorbing-desorbing process of the PAO hydrogel membrane. (Elution solution of 1.0 M Na_2CO_3 and 0.1 M H_2O_2. All scale bars in photos are 1.0 cm.). 21

Figure S17. (a) Illustration of continuous flow-through U-adsorption system containing 12 parallel adsorbent beds from natural seawater. (b) The structure and usage of the adsorbent bed. 22

Table S1. Concentration of UO_2^{2+} and co-existing metal ions in seawater and 100× seawater. 23

Table S2. Feed compositions and transformation rates (TR) from reactants to PAO hydrogels. 23

Table S3. Mechanical properties of original PAO hydrogel (m_{AAM}/m_{PAO} = 4 : 4) and adsorption-desorption cycled hydrogels. 24

Table S4. Swelling ratio (SR) and water absorptivity (WA) of PAO hydrogel (m_{AAM}/m_{PAO} = 4 : 4). 24

Table S5. Uranium adsorption capacity and elution efficiency in five adsorption-desorption cycles. 25

Supporting Movies 25

Movie S1. Fabricating process of PAO hydrogel membranes through sunlight polymerization. 25

Movie S2. U-adsorption process of PAO hydrogel membrane in 100 ppm U-spiked ultrapure water. 25

Movie S3. U-desorption process of U-uptake PAO hydrogel membrane in elution. 25

References 25

Experimental Section

Materials

Polyacrylonitrile (PAN, 99%, Mw ≈ 150 000), Hydroxylamine hydrochloride ($NH_2OH{\cdot}HCl$, 99%), Dimethy-lformamide (DMF, 99.9%), Sodium hydroxide (NaOH, 99.5%), Arsenazo (III) (95%) and Sodium Carbonate (Na_2CO_3, 99.8%) were phrased from Macklin. Acrylamide (AAM, 99.9%), 2,2'-Azobis(2-methylpropionamidine) dihydrochloride (AIBA, 99%) and N,N'-Methylenebisacrylamide (BIS, 99%) were phrased from Accelerating Scientific and Industrial. C_2H_5OH (99.5%) and Hydrochloric Acid (12 mol/L) were phrased from Xilong Scientific Co. Ltd. Uranium hexahydrate Nitrate [$UO_2(NO_3)_2{\cdot}6H_2O$, 99%] was phrased from Chushengwei Chemical Co., Ltd. All chemicals above were used as received without further purification. The natural seawater was collected from the Qiongzhou strait close to the Hainan Island side in China's Hainan province. All seawater was used after filtrated with 0.45 μm filter membrane.

Characterization

FT-IR spectra were conducted on a Perkin-Elmer LR-64912C (FT-IR, LR 64912C, Perkin-Elmer, U.S.A). UV-Vis absorption spectra were obtained with a spectrophotometer (UV1800PC, AuCy Instrument, China). Elemental electron binding energy were carried out on X-ray photoelectron spectroscopy (XPS, Thermo escalab 250Xi Thermo, U.S.A). Positional state of hydrogen and carbon studies was carried out on Nuclear magnetic resonance (NMR, Bruker AVANCE III 600M, Bruker, Germany). The microscopic morphology and structure of the hydrogels are observed and photographed on a field emission scanning electron microscope (SEM, S-4800, Hitachi, Japan). The pH values were detected via a pH meter (F2, Mettler Toledo, Germany). Adsorption selectivity of PAO hydrogel on the uranyl ion and other metal ions in a simulated seawater was studied on inductively coupled plasma mass spectrometer (ICP-OES: ICPOES730; ICP-Mass: Agilent 7500ce: Agilent, U.S.A). The Uranium adsorption capacity of the PAO hydrogel in natural seawater was measured through the other ICP-Mass (Thermoscientific iCAP RQ). The polymer molecular weight was tested through Matrix-assisted laser desorption ionization time-of-flight mass spectrometer (MALDI-TOFMS, Bruker Daltonics, Bruker, Germany). Intensities of UV-light were measured on a UV power meter (LH-126, Yongqi Electronic Equipment Co., Ltd, China). The mechanical properties of hydrogels were conducted through a tensile tester (Instron 5567).

Method of detecting uranium element concentration

Arsenazo (III) is commonly used as a uranium reagent, which can coordinate with the uranyl ion in aqueous solution. When the complex was detected by UV-Vis spectra, a specific absorption peak appears at 652 nm, and the absorbance is linear with the concentration of uranyl ion in a certain range.

Pre-configured standard uranium-spiked ultrapure-water solutions, with uranium concentrations of 0 ppm, 4 ppm, 8 ppm, 12 ppm, 16 ppm, 20 ppm, 24 ppm, 28 ppm, 32 ppm, 36 ppm, 40 ppm, 44 ppm, 48 ppm, 52 ppm, 56 ppm, 60 ppm, 64 ppm, 68 ppm, 72 ppm, 76 ppm, 80 ppm, 84 ppm, 88 ppm, 92 ppm, 96 ppm, 100 ppm standards, respectively. Pre-configured standard uranium-spiked seawater solutions with concentrations of 0 ppm, 4 ppm, 8 ppm, 12 ppm, 16 ppm, 20 ppm, 24 ppm, 28 ppm, 32 ppm, respectively. The 0.5 mL standard ultrapure-water or seawater solution was then reconstituted through adding 0.5 mL 0.1 mol/L HCl, 1.0 mL 500 mg/L arsenazo aqueous solution and 3.0 mL ultrapure water. The absorbance of each standard test solution was then measured at a fixed wavelength of 652 nm, and finally the concentration-absorbance was linearly fitted. As a result, we can achieve a curvilinear regression of concentration-absorbance for detecting the concentration of uranium in an unknown ultrapure-water (Figure S1) or seawater solution (Figure S2). After added 0.5 mL 0.1 mol/L HCl, 1.0 mL 500 mg/L arsenazo (III) aqueous solution and 3.0 mL ultrapure water into a 0.5 mL test solution, the absorbance is measured at the fixed wavelength of 652 nm through a UV-Vis spectrophotometer. The uranium concentration is determined according to the above standard curves.

We compared the two detecting uranium concentration methods between UV-Vis spectra with Arsenazo (III) and the ICP-Mass. The measuring results of uranium concentratioin through UV-Vis spectra with Arsenazo (III) are in line with the results measured through ICP-Mass. (Figure S3). Additionally, we also compared the uranium adsorption capacities of PAO hydrogel in 8 ppm U-uptake seawater and in 8 ppm simulated seawater (prepared with water contain 193 ppm sodium bicarbonate; 25,600 ppm sodium chloride; and 8 ppm uranium according to the Oak Ridge National Laboratory. The uranium adsorption capacity of PAO hydrogel in our 8 ppm U-spiked seawater is slightly lower but close to the uranium adsorption capacity in ORNL simulated seawater (Figure S4).

Test of uranium adsorption capacity

A hydrogel membrane adsorbent containing 10 mg dry matter was put into a 1.0 L uranium-spiked ultrapure-water or seawater solutions. After shaking for some time with a table concentrator, we can measure the change of uranium concentration in the solution. The uranium adsorption content of the adsorbent is obtained according to the following formula (1):

$$m_u = (C_o - C_t) \times V \qquad (1)$$

Where m_u is the mass of adsorbed uranium, C_o is the original concentration of uranium, C_t is the concentration of uranium at specific time, V is the volume of uranium-containing solution. The uranium adsorption capacity of the hydrogel membrane and PAO can be calculated through the following formula (2) and formula (3) respectively:

$$Q_{hydrogel} = m_u / m_{dry\ gel} \quad (2)$$

$$Q_{PAO} = m_u / m_{PAO} \quad (3)$$

Test of uranium desorption rate

The method used to elute uranium is based on published literature.[3] Eluent formulation: 500 ml ultrapure water, 5.7 ml 30 % aqueous hydrogen peroxide solution, 53 g sodium bicarbonate powder. Next, soak the U-uptake hydrogel membrane in the eluent at room temperature, magnetic stirring for 35 min. After the elution is complete, the hydrogel membrane is removed from the eluent and immersed in pure water, and the water is changed several times until the pH is approximately equal to 7. Finally, the hydrogel was soaked in the uranium-spiked seawater solution (32 ppm) for the next cycle. Repeat this adsorption-elution process for five times.

Synthesis of PAO

The PAO was synthesized on the basis of the reported literature.[1,2] After the $NH_2OH{\cdot}HCl$ (5.56 g, 80 mmol) was dissolved in DMF (60 mL) within a round-bottom flask at 45 ℃, the Na_2CO_3 (3.82 g, 36 mmol) and NaOH (0.96 g, 24 mmol) was added and stirred by magnetic force for at least 3 h; dissolved PAN (4.24 g, 80 mmol) at 45 ℃, and then reacted at 65 ℃ for 24 h; replenished Na_2CO_3 (1.91 g, 18 mmol) and NaOH (0.48 g, 12 mol), and then reacted at 65 ℃ for 12 h. The reaction mixture was dropped into deionized water to get a white flocculent precipitate. After filtered by a bush funnel, the precipitate was dried in a vacuum at 60 ℃ for 8 h to achieve as-prepared PAO.

Fabrication of PAO Semi-IPN hydrogels

Seven PAO semi-IPN hydrogels were prepared with different mass ratios of AAM and PAO (Figure S9, Movie S1). Firstly, we dissolved monomer AAM (40 mg), cross-linker (6 mg), initiator AIBA (6 mg) and PAO (0 mg, 20 mg 30 mg, 40 mg 50 mg, 60 mg 0 mg, 80 mg with different mass ratios of AAM and PAO 4 : 0, 4 : 2, 4 : 3, 4 : 4, 4 : 5, 4 : 6 and 4 : 8, respectively) in 0.3 mol/L NaOH (1 mL) aqueous solution; after ultrasound treatment was conducted for 5-10 minutes, the precusor solution was transfer into a 0.2 mm thickness reaction tank and sealed; then the precursor solution was polymerized for 15 min under UV LED lamp (with a UV intensity of 2.2 ± 0.1 mW/cm^2), the PAO Semi-IPN hydrogel membranes can be prepared. Furtherly, the sunlight (with a UV intensity of 2.5 ± 0.2 mW/cm^2 for 10

min) can replace the UV lamp well, and all the hydrogel membranes used in the characterization and testing process were fabricated by this kind of sunlight-polymerization.

Test of hydrogel adsorption selectivity on different ions

The simulated seawater was prepared for testing the hydrogel adsorption selectivity. Methods in this experiment were obtained according to the National Standard of the People's Republic of China (HY/T 147.1-2013). In this simulated seawater, the concentration of Na, Ca, Mg and K is equal to the natural seawater; the concentration of U, V, Cu, Fe, Ni and Zn is as 100 times as the natural seawater. After immersed the PAO hydrogel membrane (m_{AAM} : m_{PAO} = 4 : 4, 10 mg dry weight) in the simulated seawater (1000 mL) for 48 h, each metal ion with high-concentration was measured through ICP-OES (for Na, Ca, Mg and K), and each metal ion with low concentration was measured through ICP-Mass (for U, V, Cu, Fe, Ni and Zn). The adsorption of different ions can be calculated through analyzing the concentrations before and after adsorption. The adsorption selectivity of the PAO hydrogel on U and other co-existing metal ions can be indicated through comparing the different adsorption capacities of them.

Test of hydrogel uranium adsorption from natural seawater

As shown in Figure S17a, the continuous flow-through U-adsorption system containing 12 parallel adsorbent beds. The hydrogel membrane can be sandwiched by the two sponges (Figure S17b). We can place 12 pieces PAO hydrogel membranes (m_{AAM}/m_{PAO} = 4 : 4, each piece owning 3 mg of dry mass) in the 12 beds, respectively. The flowing speed of seawater through each adsorbent bed can be independently controlled about 1.5 L/min. In 4 weeks, took seawater samples (200 mL at a time) and replaced the old sponge with new one once a week. The uranium adsorption capacity of hydrogel samples from seawater can be measured through the ICP-Mass (Thermoscientific iCAP RQ).

Data analysis

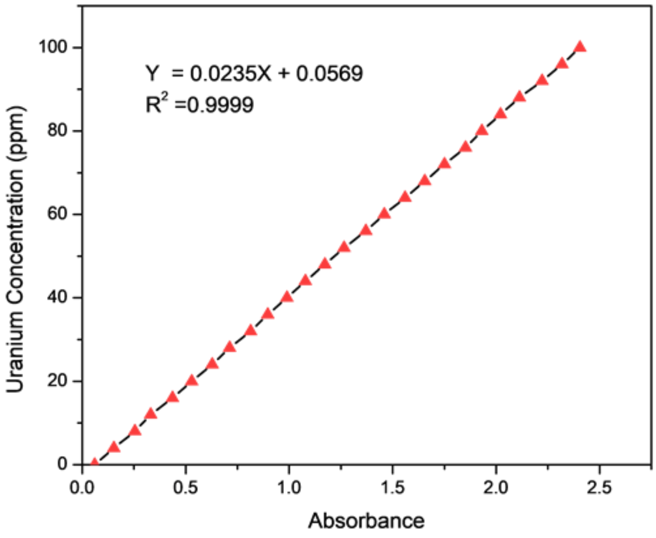

Figure S1. The curvilinear regression of uranium concentration-absorbance in uranium-spiked ultrapure water.

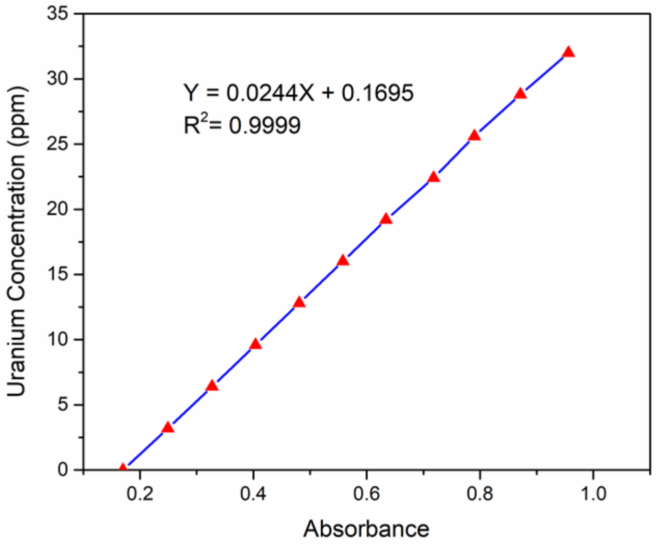

Figure S2. The curvilinear regression of uranium concentration-absorbance in uranium-spiked natural seawater.

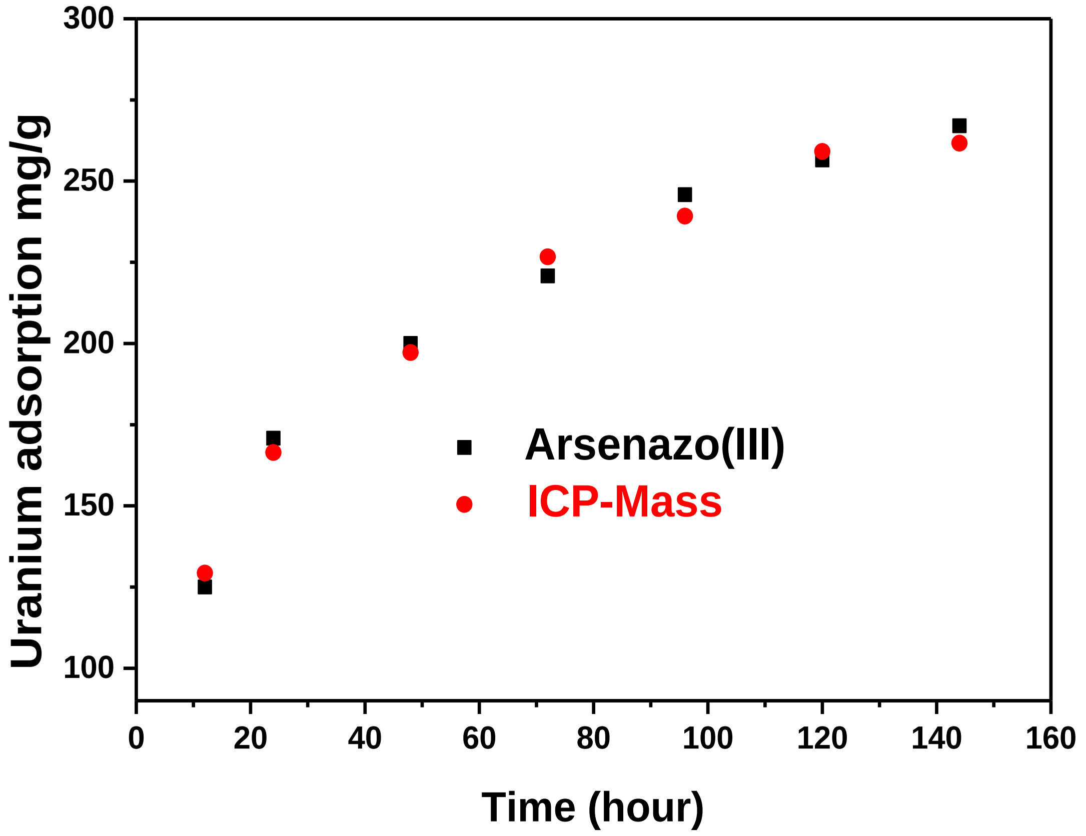

Figure S3. Comparison of the Arsenazo (III) method and ICP-Mass method for measuring uranium concentration (with a PAO hydrogel owning 10 mg dry mass immersed in 1000 mL 8 ppm uranim-spiked seawater).

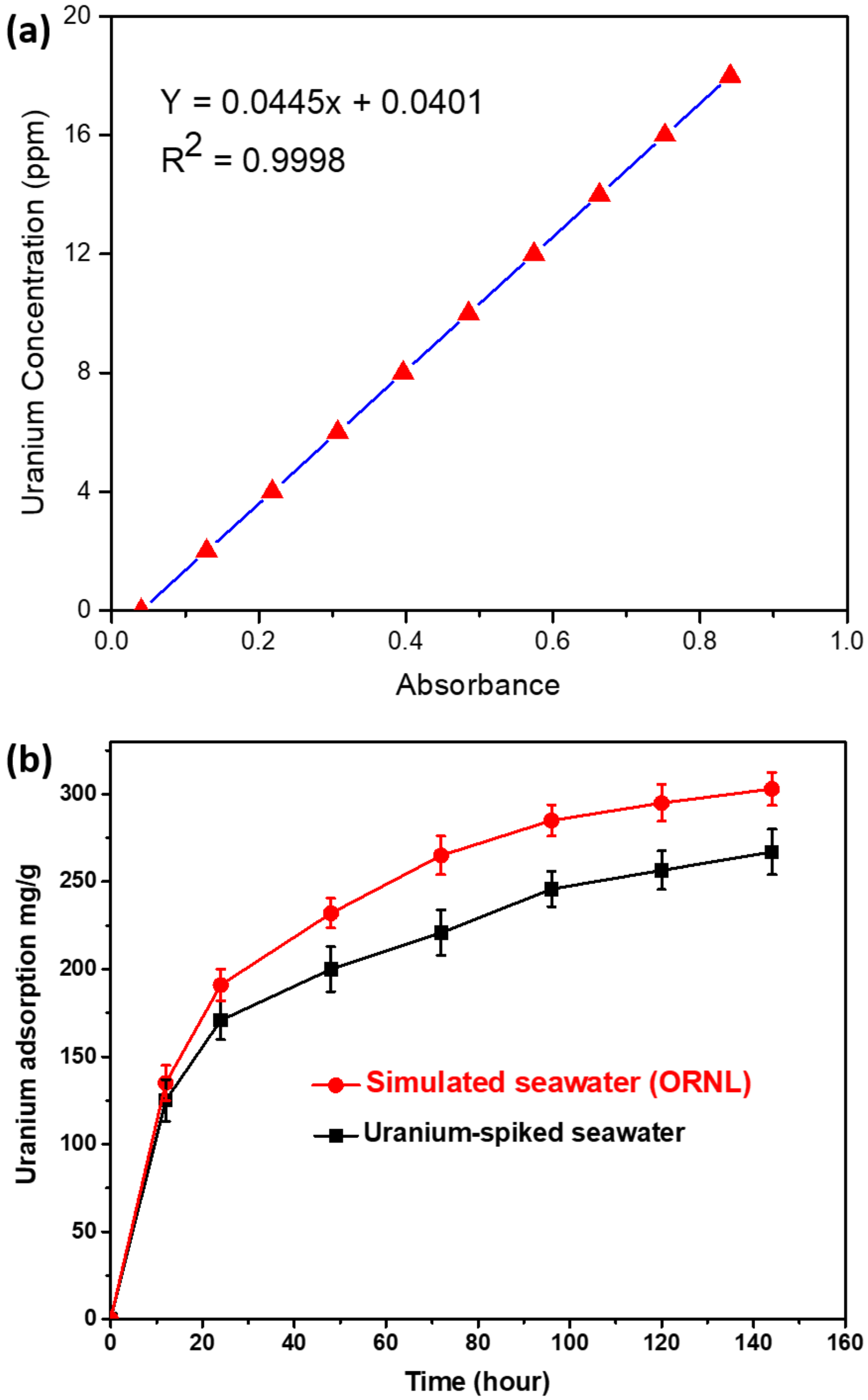

Figure S4. (a) The curvilinear regression of uranium concentration-absorbance in simulated seawater. (b) Comparison of uranium adsorption capacities between PAO hydrogel (m_{AAM} : m_{PAO} = 4 : 4) in 8 ppm U-uptake seawater and in simulated seawater. (The simulated seawater was prepared with water contain 193 ppm sodium bicarbonate; 25,600 ppm sodium chloride; and 8 ppm uranium according to the Oak Ridge National Laboratory.)

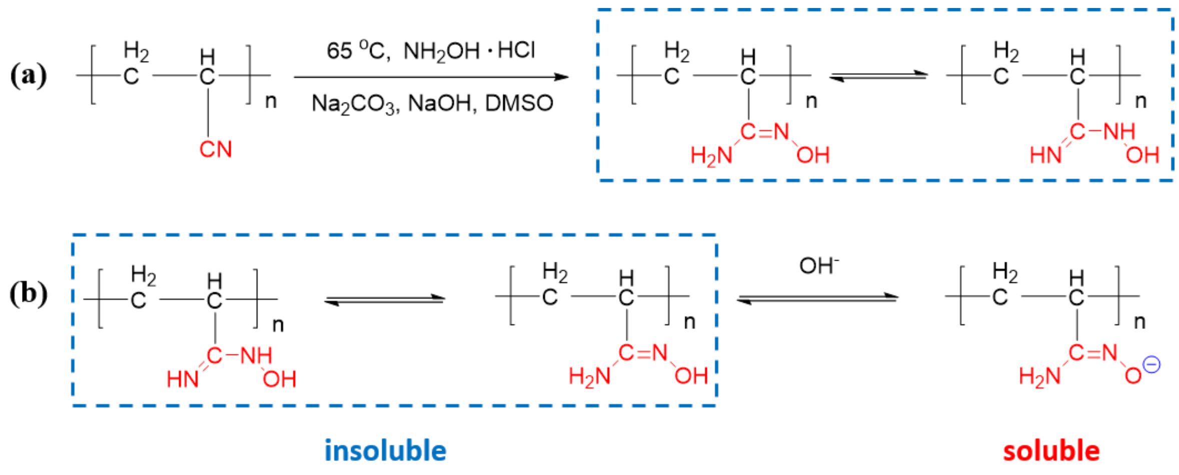

Figure S5. (a) Synthesis of water-insoluble PAO; (b) Synthesis of water-soluble PAO salt.

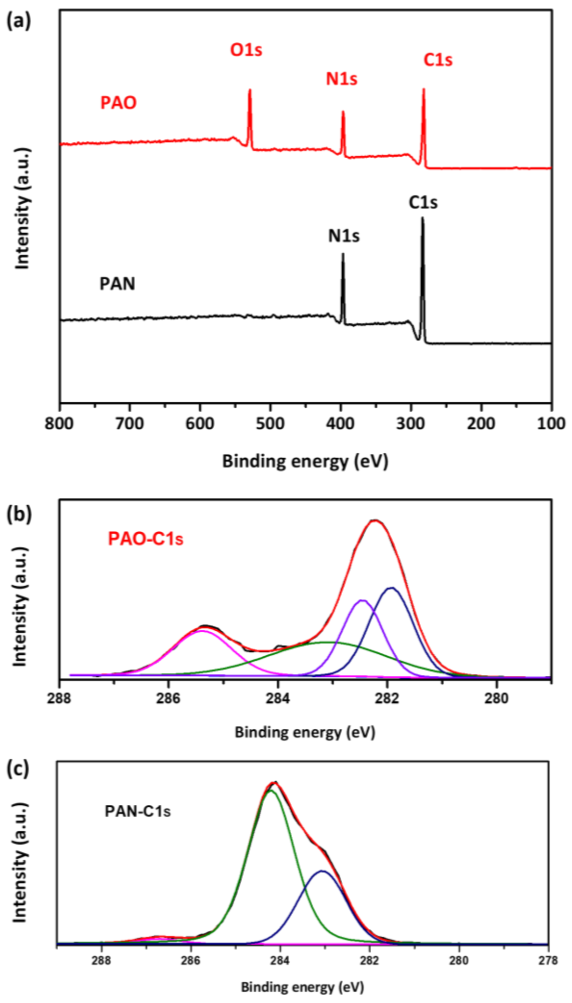

Figure S6. XPS spectra of PAN and PAO. (a) Survey XPS spectra of PAN and PAO; (b) and (c) are the high-resolution XPS spectra of the PAN and PAO, respectively.

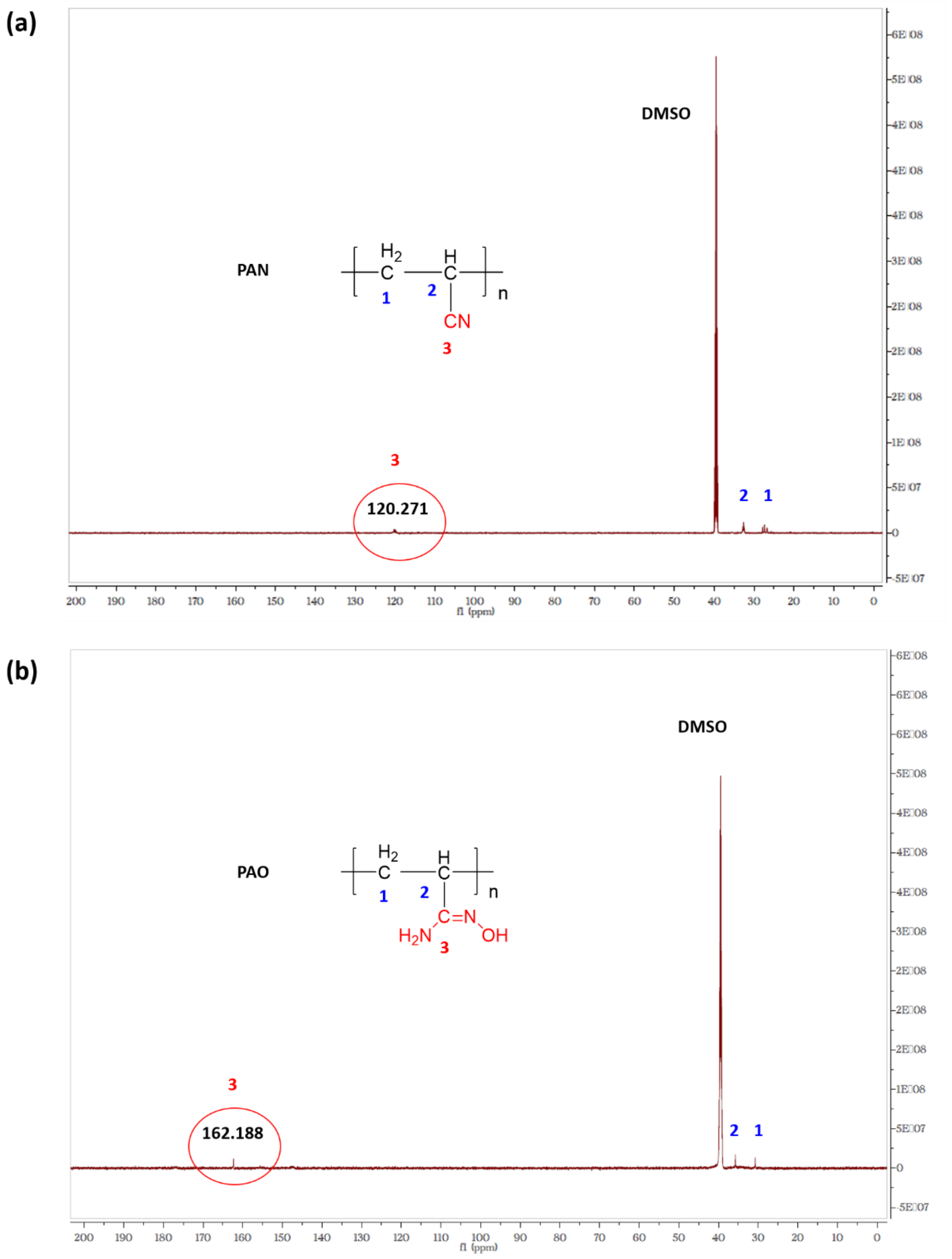

Figure S7. The ^{13}C-NMR spectra of (a) PAN and (b) PAO in DMSO-D_6, respectively.

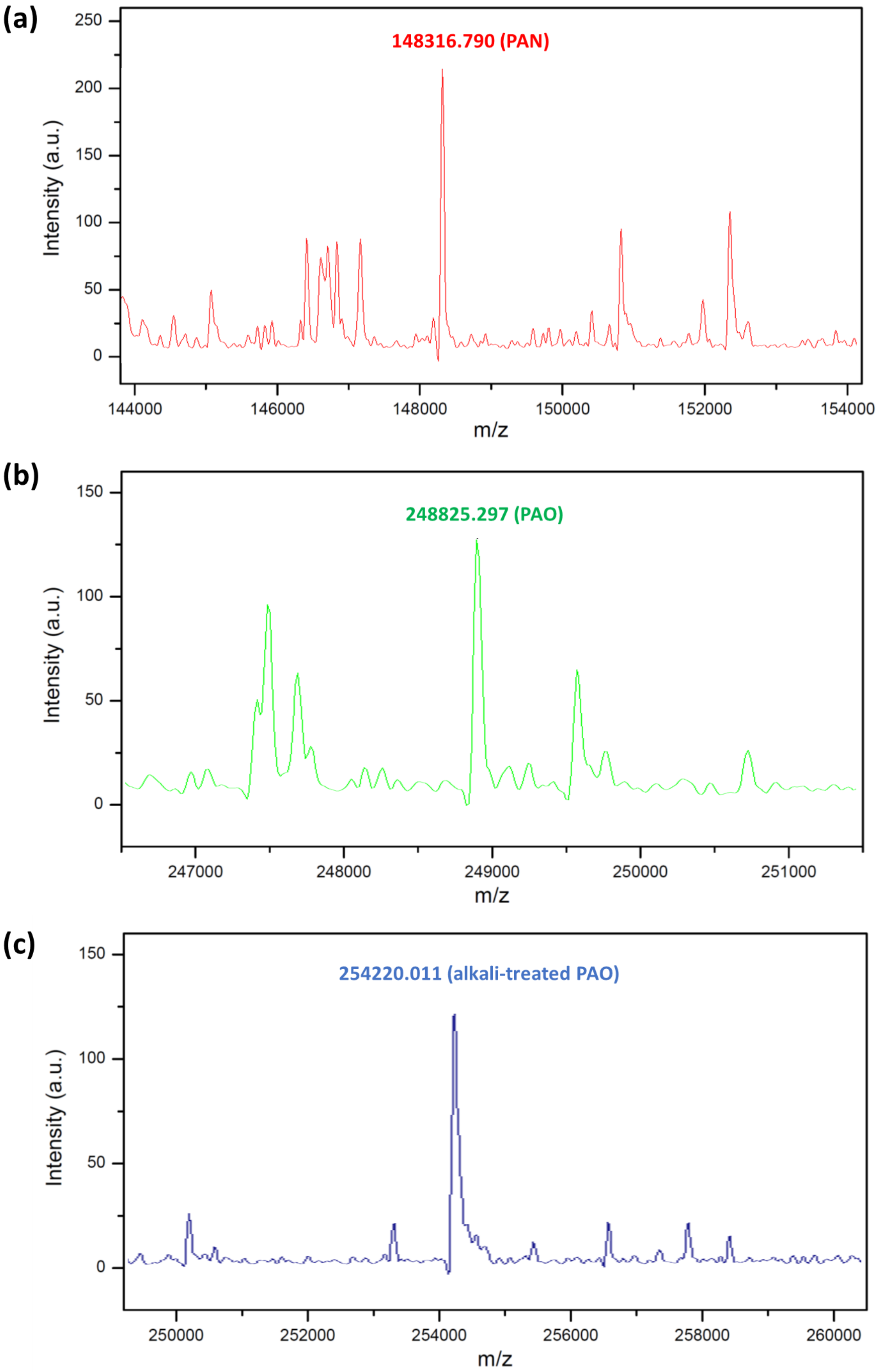

Figure S8. Time-of-flight MS of (a) PAN, (b) PAO, (c) alkali-treated PAO), respectively.

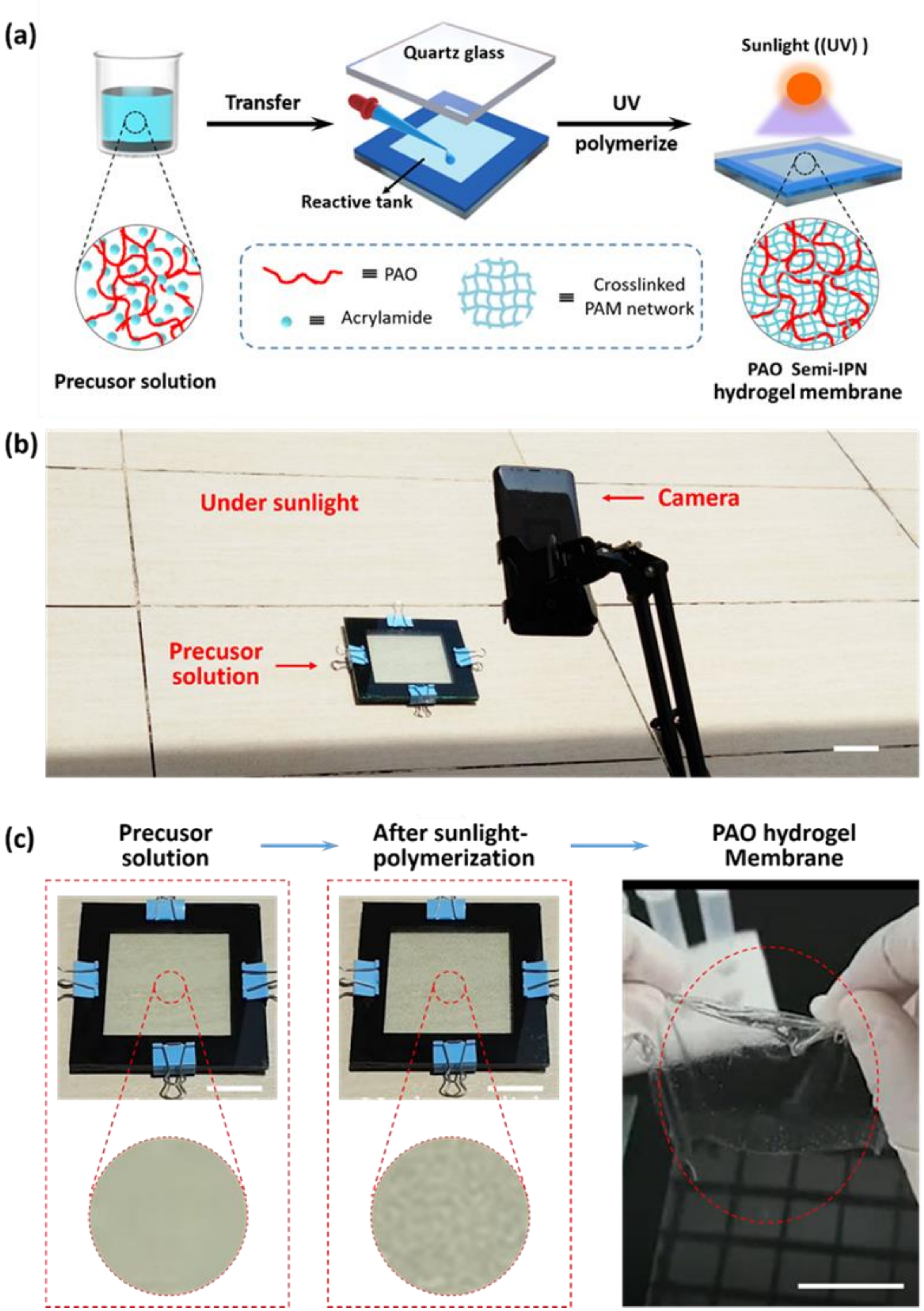

Figure S9. Process of massively and directly fabricating of PAO hydrogel membrane. (a) Illustration of fabricating PAO hydrogel and the Semi-IPN hydrogel structure. (b) Massively and directly producing the PAO hydrogel membrane in sunlight. (c)The self-supporting PAO hydrogel membrane. (All scale bars in photos are 5.0 cm)

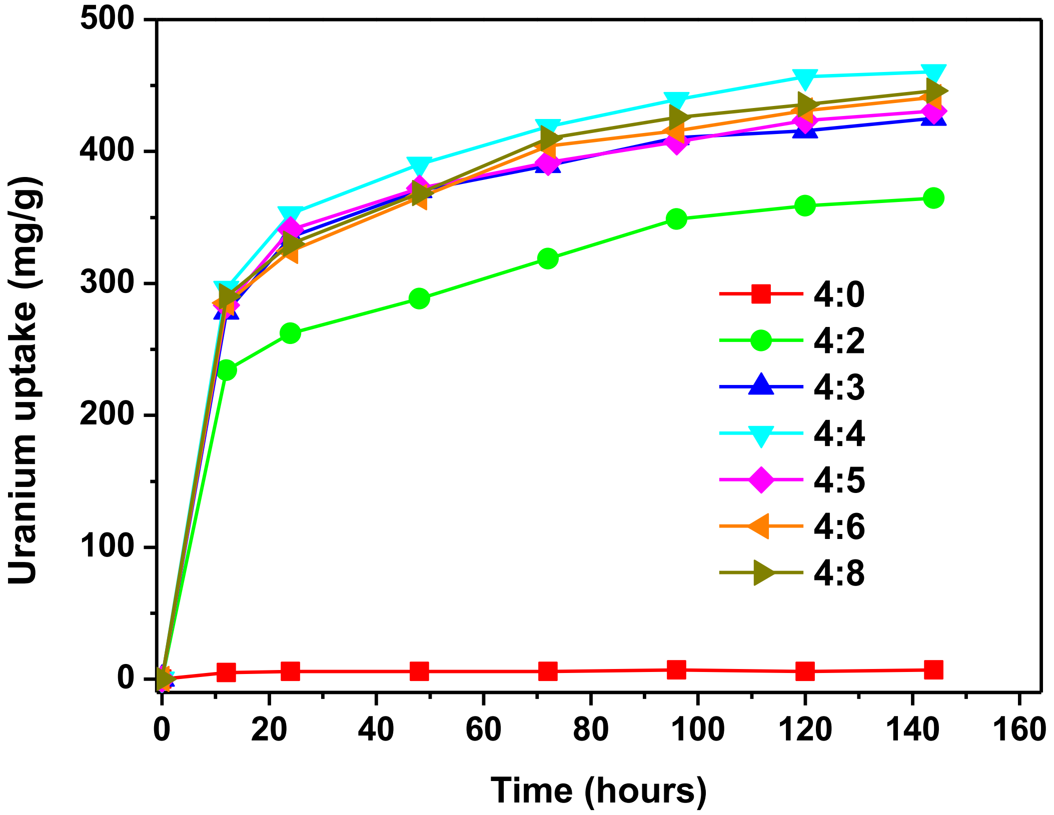

Figure S10. Uranium adsorption kinetics of PAO hydrogel membranes with different mass ratios of PAO/AAM in 32 ppm uranium-spiked natural seawater.

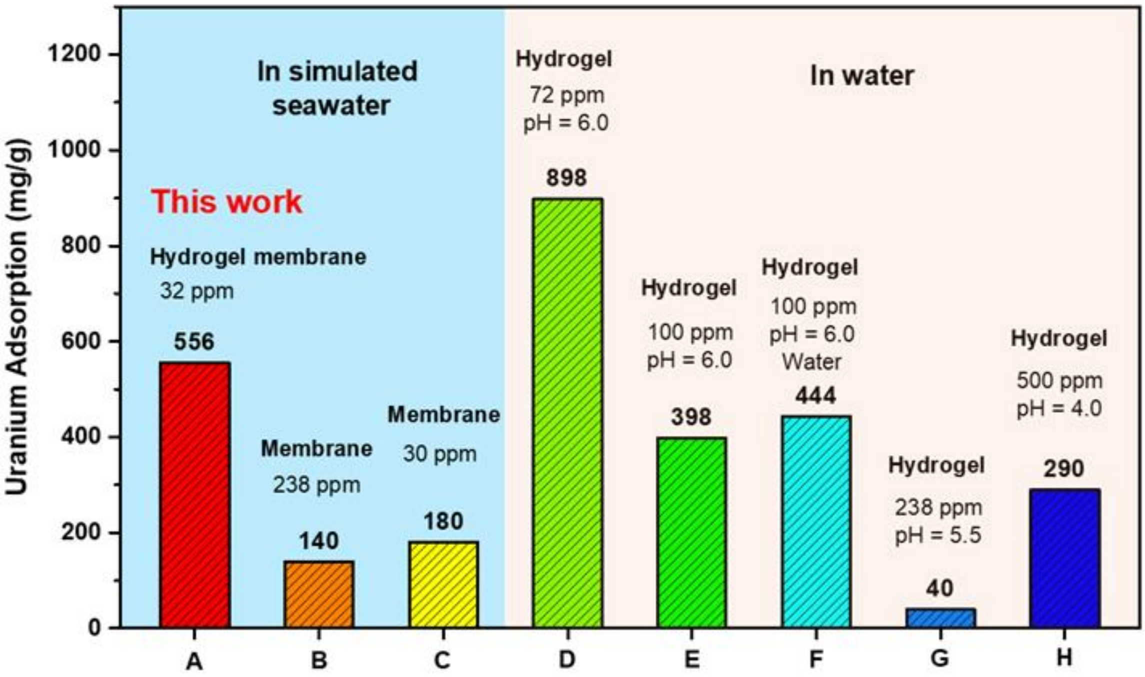

Figure S11. Comparison of adsorption capacities among hydrogel-based and membrane-based adsorbents [The adsorption capacity of sample A is our PAO hydrogel (m_{AAM}/m_{PAO} = 4 : 4), the other adsorption capacities of B-G samples were cited from published articles.[4-9]].

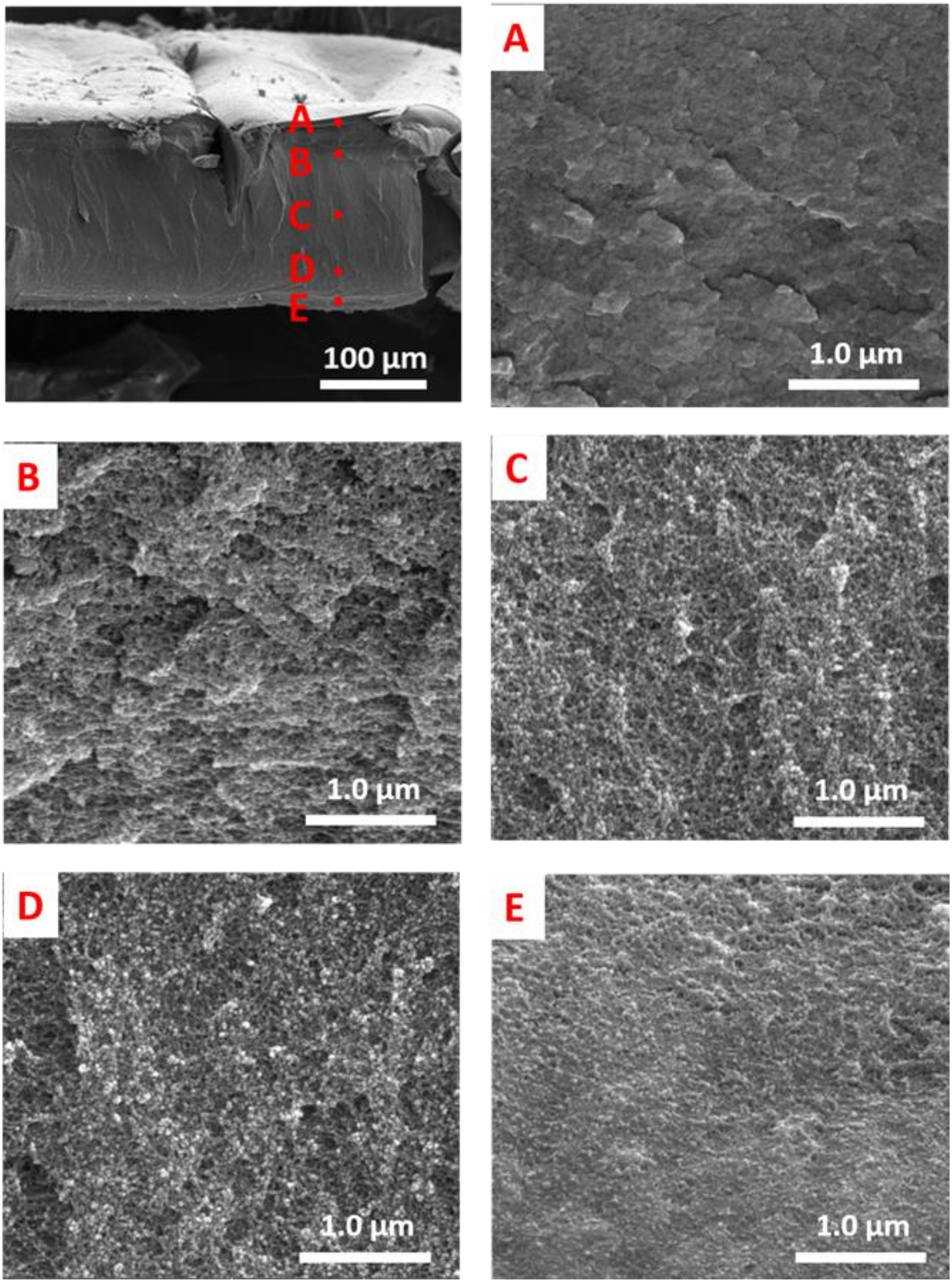

Figure S12. SEM images of surface and inner part of U-uptake PAO hydrogel (m_{AAM}/m_{PAO} = 4 : 4, 312 mg/g).

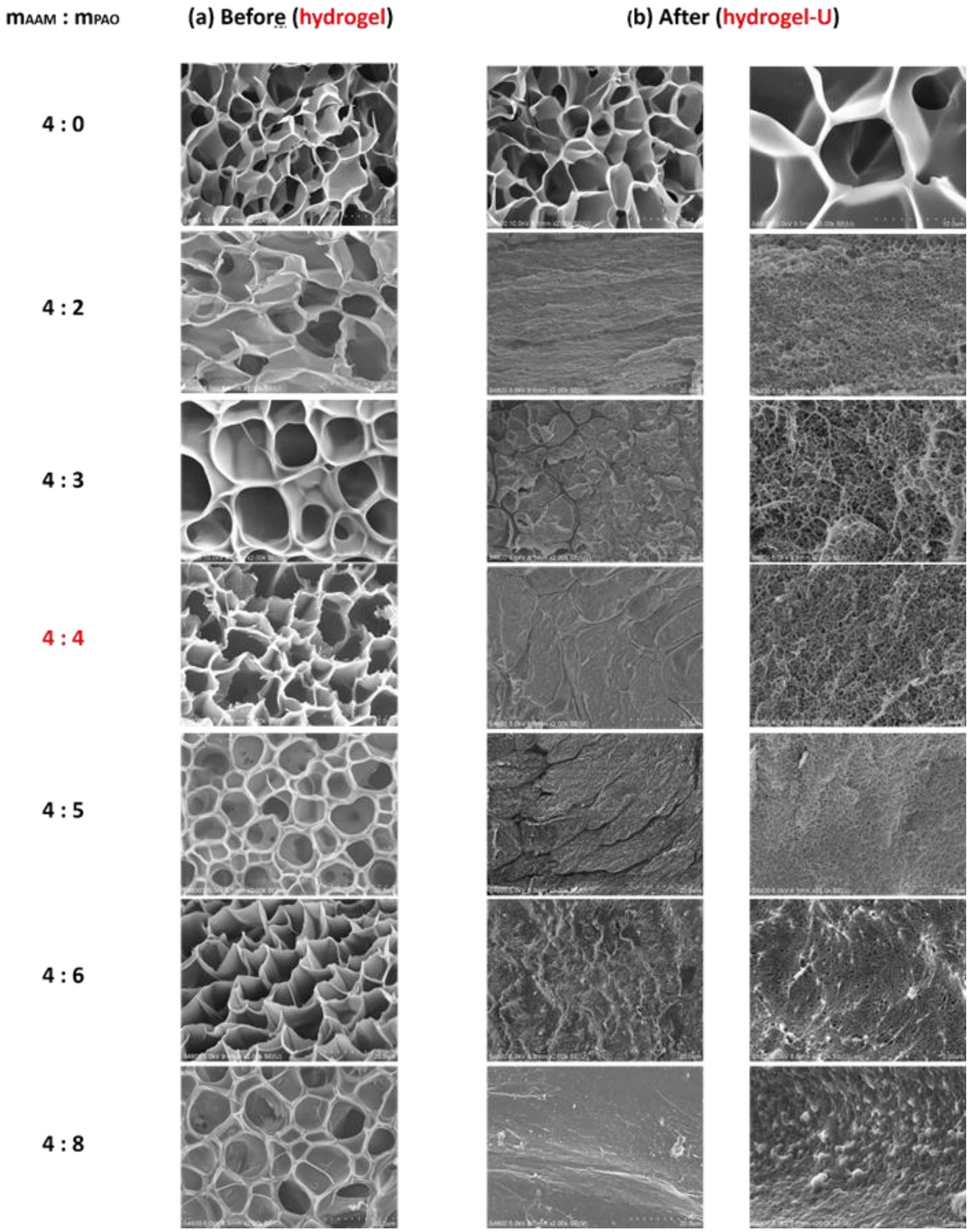

Figure S13. SEM images of PAO hydrogels (a) before and (b) after Uranium adsorption with different mass ratios of PAO/AAM.

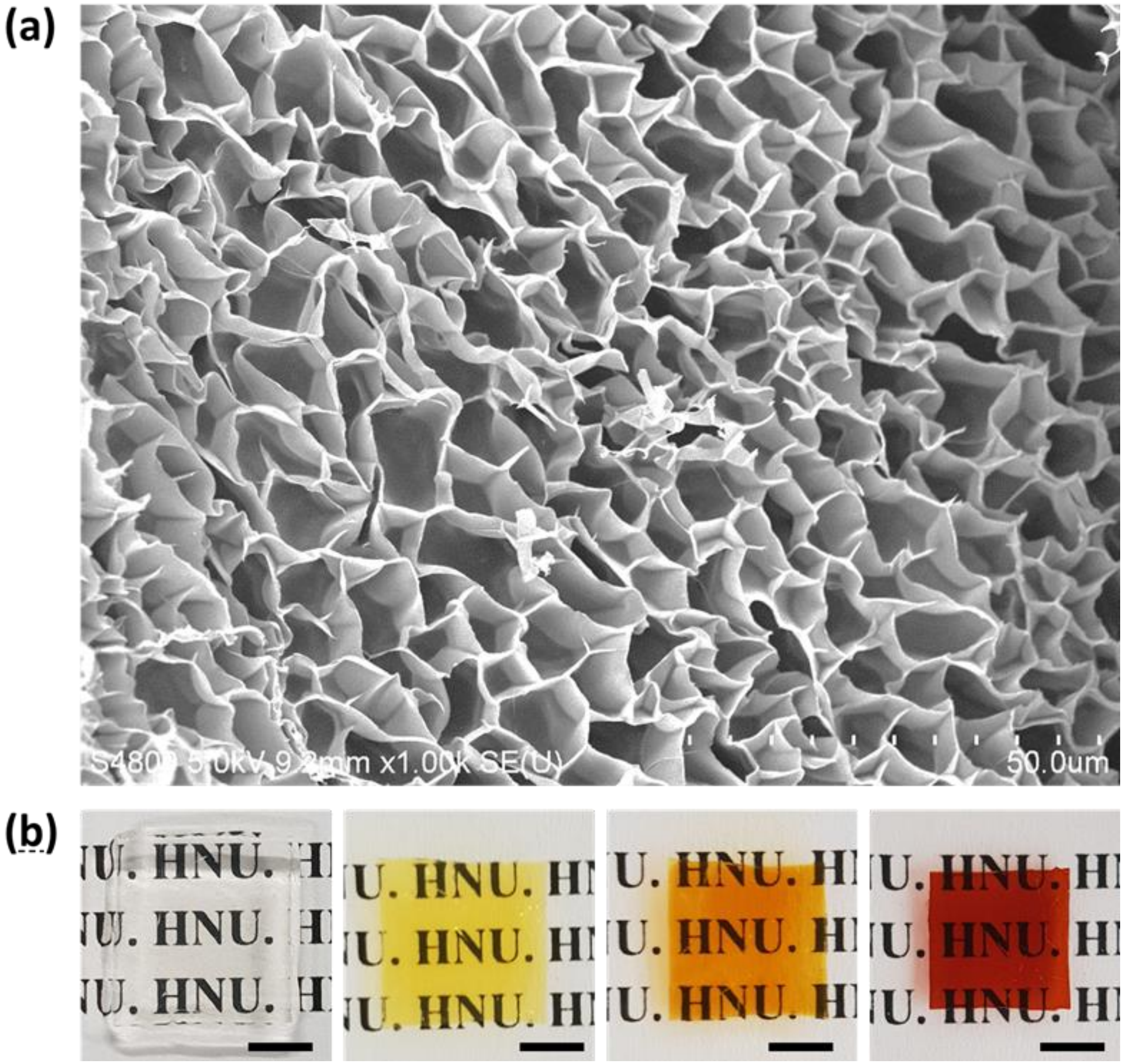

Figure S14. (a) The SEM image of original PAO hydrogel (m_{AAM} : m_{PAO} = 4 : 4). (b) The transparent property of U-uptake PAO hydrogels (All scale bars in photos are 1.0 cm.).

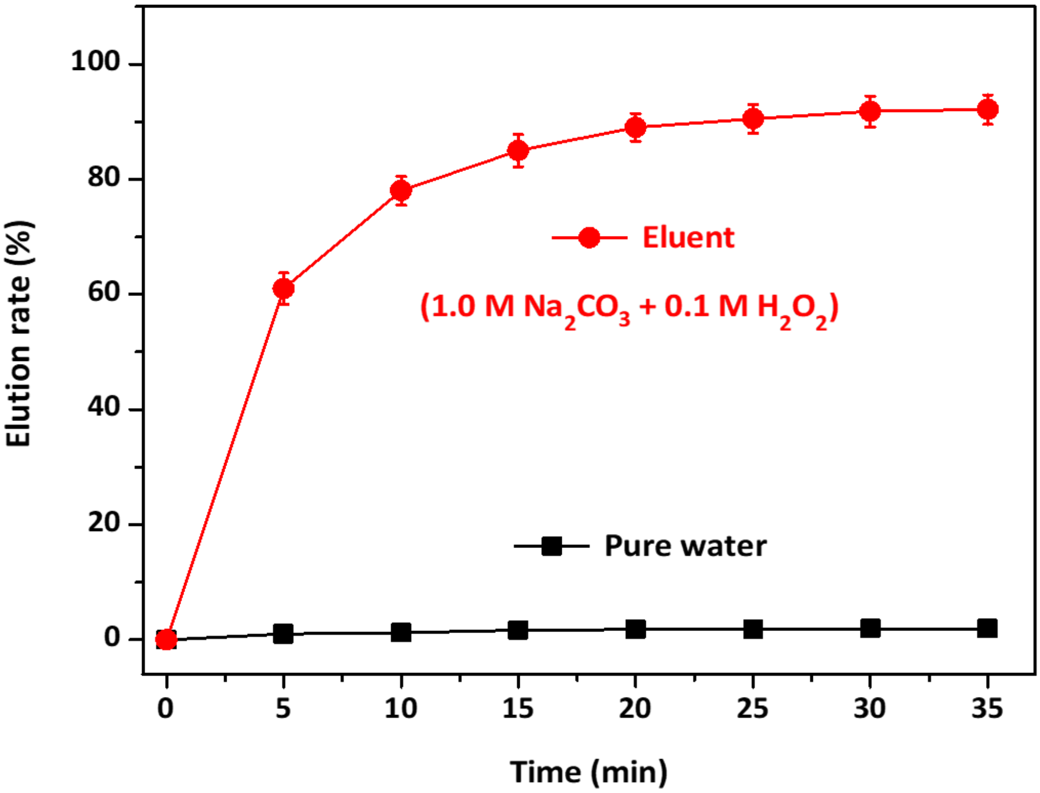

Figure S15. Uranium desorption kinetics of PAO hydrogel membranes in the elution solution.

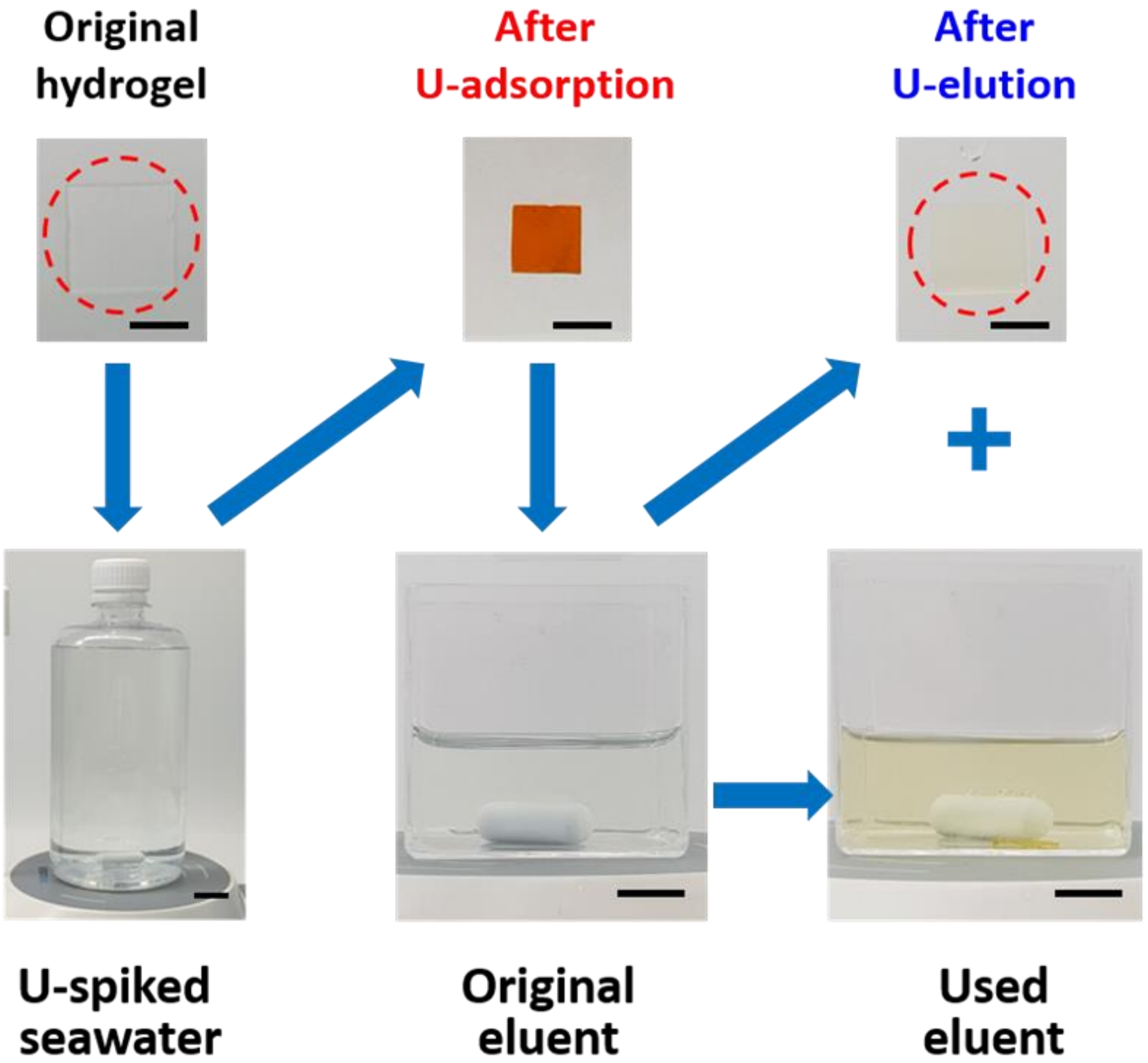

Figure S16. The uranium adsorbing-desorbing process of the PAO hydrogel membrane. (Elution solution of 1.0 M Na_2CO_3 and 0.1 M H_2O_2. All scale bars in photos are 1.0 cm.)

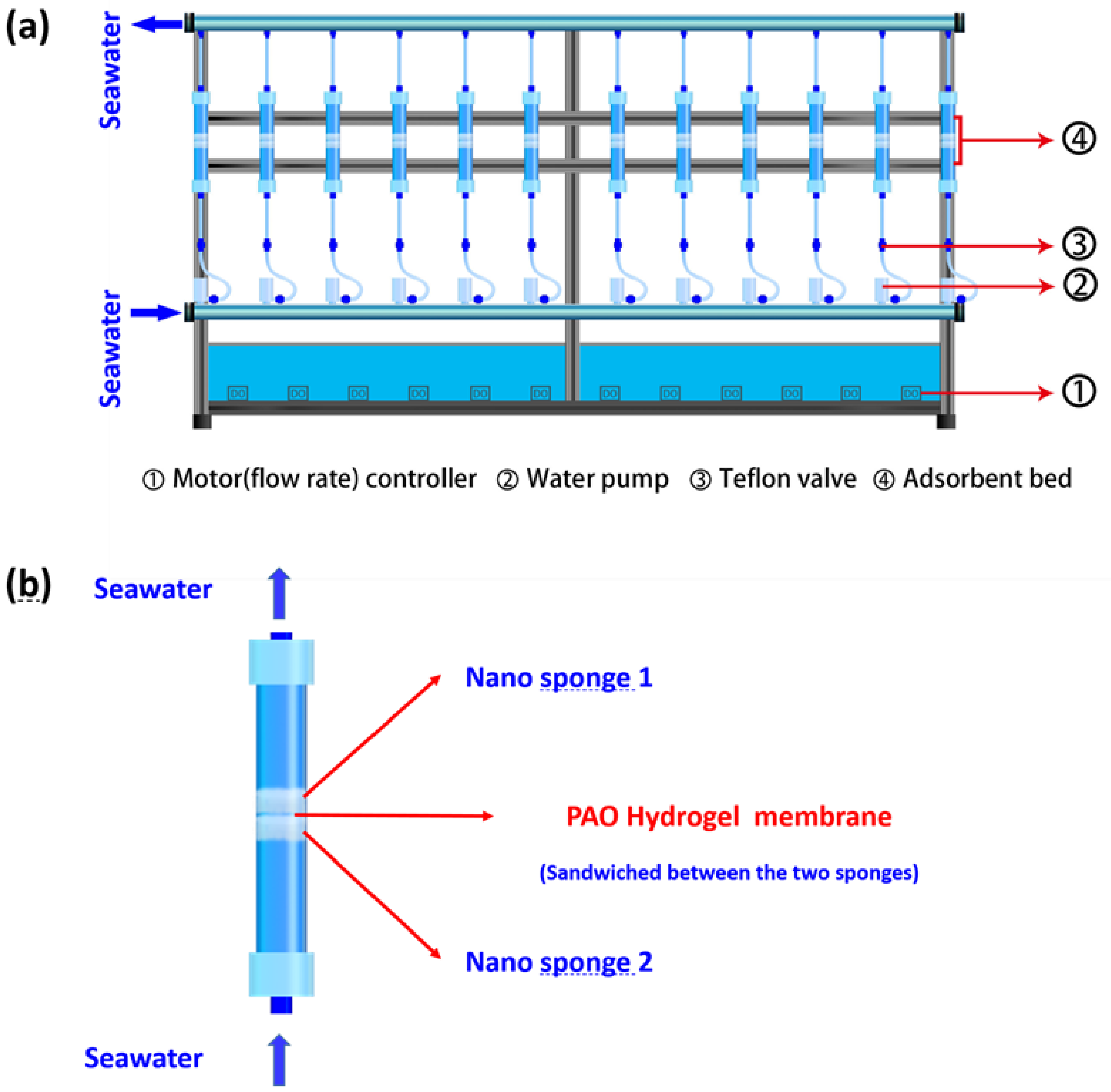

Figure S17. (a) Illustration of continuous flow-through U-adsorption system containing 12 parallel adsorbent beds from natural seawater. (b) The structure and usage of the adsorbent bed.

Table S1. Concentration of U(VI) and co-existing metal ions in seawater and 100× seawater.

Element	Con. In natural SW/ppb	Con. In 100× SW(calculated)/ppb	Ions
U	3.3	330	U(VI)
V	2.6	260	V(V)
Ni	0.9	90	Ni^{2+}
Cu	2.8	280	Cu^{2+}
Fe	1.7	1.7	Fe^{3+}
Zn	1.05	1.05	Zn^{2+}
Na	10.26×10^{6}	10.26×10^{6}	Na^{+}
K	0.65×10^{6}	0.65×10^{6}	K^{+}
Ca	0.92×10^{6}	0.92×10^{6}	Ca^{2+}
Mg	1.22×10^{6}	1.22×10^{6}	Mg^{2+}

Table S2. Feed compositions and transformation rates (TR) from reactants to PAO hydrogels.

	Hydrogel Samples with different m_{AAM}/m_{PAO}						
	4 : 0	4 : 2	4 : 3	4 : 4	4 : 5	4 : 6	4 : 8
AAm (mg)	40	40	40	40	40	40	40
PAO (mg)	0	20	30	40	50	60	80
BIS (mg)	6	6	6	6	6	6	6
AIBA (mg)	6	6	6	6	6	6	6
Ionic water (mL)	1.0	1.0	1.0	1.0	1.0	1.0	1.0
TR (%) [a]	99.4	98.7	99.3	98.8	99.2	98.9	99.3

a: TR = $[m_{(dry\ gel)}/m_{(Feed\ compositions)}] \times 100\%$

Table S3. Mechanical properties of original PAO hydrogel (m_{AAM}/m_{PAO} = 4 : 4) and adsorption-desorption cycled hydrogels.

Sample [a]	Strength at break (kPa)	Yang's modulus (kPa)	Elongation at break (%)
Original PAO hydrogel	54.4 ± 3.9	137 ± 6.3	38.3 ± 7.7
Hydrogel after 1 cycle	57.3 ± 4.6	141 ± 5.8	35.5 ± 7.1
Hydrogel after 2 cycles	55.8 ± 5.1	139 ± 4.7	37.8 ± 8.3
Hydrogel after 3 cycles	53.8 ± 6.5	142 ± 5.5	35.7 ± 6.9
Hydrogel after 4 cycles	56.7 ± 6.0	138 ± 6.4	34.2 ± 6.7
Hydrogel after 5 cycles	57.8 ± 5.6	140 ± 5.2	36.2 ± 8.1

a: All the PAO hydrogel samples with a dumbbell shape (length: 60 mm, width: 10 mm, thickness: 0.2 mm) were prepared for the tensile testing. All the tests were measured at a constant rate of 50 mm/min.

Table S4. Swelling ratio (SR) and water absorptivity (WA) of PAO hydrogel (m_{AAM}/m_{PAO} = 4 : 4).

Sample	SR[a] (%)	WA[b] (%)
Original PAO hydrogel (in 0.3 M NaOH)	2183 ± 87	2083 ± 87
Original PAO hydrogel (in water)	1408 ± 68	1308 ± 68
Original PAO hydrogel (in seawater)	1112 ± 56	1012 ± 56

a: SR = $[m_{hydrogel}/m_{dry\ gel}] \times 100\%$
b: WA = $[m_{hydrogel}-m_{dry\ gel}]/\ m_{dry\ gel} \times 100\%$

Table S5. Uranium adsorption capacity and elution efficiency in five adsorption-desorption cycles.

Cycle number	$Q_{hydrogel}$ [m_U (mg)/ $m_{dry\ gel}$ (g)]	Q_{PAO} [m_U (mg)/ m_{PAO} (g)]	Elution efficiency (%)
1	444.7 ± 4.51	811.7 ± 10.4	95.35 ± 4.29
2	415.8 ± 6.02	759 ± 13.85	93.06 ± 5.95
3	391.9 ± 6.54	715.3 ± 15	91.37 ± 6.68
4	371.7 ± 7.61	678.5 ± 17.5	88.64 ± 7.94
5	340.2 ± 4.56	621 ± 10.5	86.98 ± 5.12

Supporting Movies

Movie S1. Fabricating process of PAO hydrogel membranes through sunlight polymerization.

Movie S2. U-adsorption process of PAO hydrogel membrane in 100 ppm U-spiked ultrapure water.

Movie S3. U-desorption process of U-uptake PAO hydrogel membrane in elution.

Reference：

[1] H.-B. Pan, L.-J. Kuo, C. M. Wai, N. Miyamoto, R. Joshi, J. R. Wood, J. E. Strivens, C. J. Janke, Y. Oyola, S. Das, R. T. Mayes, G. A. Gill, *Ind. Eng. Chem. Res.* **2016**, *55*, 4313.

[2] S. O. Kang, S. Vukovic, R. Custelcean, B. P. Hay, *Ind. Eng. Chem. Res.* **2012**, *51*, 6619.

[3] H.-B. Pan, W. Liao, C. M. Wai, Y. Oyola, C. J. Janke, G. Tian, L. Rao, *Dalton Trans*. **2014**, *43*, 10713.

[4] W. Luo, G. Xiao, F. Tian, J. J. Richardson, Y. Wang, J. Zhou, J. Guo, X. Liao, B. Shi, *Energy Environ. Sci*. **2019**, *12*, 607.

[5] L. Zhou, H. Zou, Y. Wang, Z. Liu, Z. Le, G. Huang, T. Luo, A. A. Adesina, J. Radioanal. *Nucl. Chem*. **2017**, *311*, 779.

[6] X. Wang, Q. Liu, J. Liu, R. Chen, H. Zhang, R. Li, Z. Li, J. Wang, *Appl. Surf. Sci.* **2017**, *426*, 1063.

[7] F. Wang, H. Li, Q. Liu, Z. Li, R. Li, H. Zhang, L. Liu, G. A. Emelchenko, J. Wang, *Sci. Rep*. **2016**, *6*, 19367.

[8] S. Su, R. Chen, Q. Liu, J. Liu, H. Zhang, R. Li, M. Zhang, P. Liu, J. Wang, *Chem. Eng. J.* **2018**, *345*, 526.

[9] F. Xiao, Y. Sun, W. Du, W. Shi, Y. Wu, S. Liao, Z. Wu, R. Yu, *Adv. Funct. Mater.* **2017**, *27*, 1702147.

[10] J. He, F. Sun, F. Han, J. Gu, Minrui Ou, W. Xu, X. Xu, *RSC Adv.* **2018**, *8*, 12684.
